# Use of the 10-meter timed walk for monitoring long-term gait progression in human T-cell leukemia virus type 1-associated myelopathy/tropical spastic paraparesis: a 10-year analysis from the Japanese HAM-net registry

**DOI:** 10.1186/s13023-026-04399-y

**Published:** 2026-05-25

**Authors:** Fuyuhiko Marubayashi, Yasuyuki Mogi, Tomoo Sato, Naoko Yagishita, Manabu Ishii, Hideaki Hida, Kenichiro Tanabe, Yoshihisa Yamano

**Affiliations:** 1 Department of Medical Science, Tanabe Pharma America, Inc., Jersey City, NJ USA; 2 Department of Rare Diseases Research, Institute of Medical Science, St. Marianna University School of Medicine, Kawasaki, Japan; 3 Department of Neurology, St. Marianna University School of Medicine, Kawasaki, Japan; 4 Department of Data Science, Division of IT Digital, Tanabe Pharma Corporation, Marunouchi Chiyoda-ku, Tokyo, Japan; 5 Department of Frontier Medicine, Institute of Medical Science, St. Marianna University School of Medicine, Kawasaki, Japan

**Keywords:** Human T-cell leukemia virus type 1 (HTLV-1), Human T-cell leukemia virus type 1-associated myelopathy/tropical spastic paraparesis (HAM/TSP), 10-meter timed walk (10mTW), Motor disability score, Activities of daily living (ADL), Real-world data (RWD)

## Abstract

**Background:**

Human T-cell leukemia virus type 1 (HTLV-1)-associated myelopathy/tropical spastic paraparesis (HAM/TSP) is a chronic neuroinflammatory disease that affects approximately 0.3% of individuals with HTLV-1 infection in Japan. Considering the rarity of HAM/TSP, data on the natural progression of walking disability and its correlation with outcome measures, such as the Osame’s Motor Disability Score (OMDS), remain limited. This observational study analyzed longitudinal data from the Japanese HAM patient registry “HAM-net” to identify appropriate clinical outcome measures for evaluating treatment efficacy in HAM/TSP.

**Methods:**

We incorporated 10-meter timed walk (10mTW) data from patients registered in the HAM-net. The natural course of 10mTW performance was assessed, and its correlation with various outcome measures was analyzed to evaluate the use of the 10mTW as a primary outcome measure in HAM/TSP clinical trials.

**Results:**

This study included 142 patients with at least two 10mTW data; 77.5% were female, with a mean age at onset and HAM diagnosis of 45.7 and 53.8 years, respectively. The geometric mean (95% confidence interval [CI]) for the 10mTW at baseline, Year 5, and Year 10 was 12.6 s (95% CI, 11.5 s–13.8 s), 14.3 s (95% CI, 12.7 s–16.1 s), and 16.3 s (95% CI, 13.6 s–19.5 s), respectively. The 10mTW at Years 5 and 10 increased by 7.37% (95% CI, −0.39%–15.73%) and 42.10% (95% CI, 19.92%–68.39%), respectively. The 10mTW exhibited significant positive correlations with motor disability scores, such as the OMDS and Instituto de Pesquisa Clinica Evandro Chagas-1 (IPEC-1), and activities of daily living (ADL) scales, such as the Health Assessment Questionnaire (HAQ).

**Conclusions:**

This study revealed a 42.10% increase in the 10mTW over 9 years, with significant correlations to motor disability scores (OMDS and IPEC-1) and ADL scales (HAQ). These findings support the use of 10mTW performance as a primary outcome measure in HAM/TSP clinical trials, emphasizing its value for long-term assessment of gait progression.

**Supplementary information:**

The online version contains supplementary material available at 10.1186/s13023-026-04399-y.

## Background

Human T-cell leukemia virus type 1 (HTLV-1)-associated myelopathy/tropical spastic paraparesis (HAM/TSP) is a chronic neuroinflammatory disease that affects approximately 0.3%–3.8% of patients with HTLV-1 infection [1–4]. This disease is characterized by persistent myelopathic symptoms, including spastic paraparesis, sensory disturbances in the lower limbs, and bladder/bowel dysfunction [5]. The estimated number of patients with HAM/TSP in Japan is approximately 3,000 [6]. Although HTLV-1 is highly prevalent in Japan, Africa, the Caribbean, and Central and South America, the actual worldwide prevalence of HAM/TSP remains unknown owing to the lack of data in most countries [7]. Given the low prevalence of HTLV-1 in economically advanced countries outside Japan, the number of patients with HAM/TSP in these countries is expected to be low [7]. Consequently, international data on biomarkers and treatment strategies are limited. Like other rare diseases, research on HAM/TSP faces challenges because of the dispersion of patient populations across multiple medical institutions. This makes consolidating comprehensive information on natural history, prognostic factors, therapeutic efficacy, and criteria for evaluating treatment outcomes difficult, all of which are crucial for developing treatments. Studies targeting Caribbean cohorts that have a high prevalence of HAM/TSP have provided important insights into the natural history of HAM/TSP. Long-term follow-up studies reported the median times from disease onset to unilateral, bilateral support and wheelchair use of 6, 13, and 21 years, respectively [8]; and 11 and 18 years to bilateral support and wheelchair use, respectively [9]. These data alone are insufficient to fully understand disease progression and management.

To address these challenges, a relatively large national registry system for patients with HAM/TSP, known as HAM-net, was established in Japan in 2012 to prospectively collect detailed data from patients with HAM/TSP. The HAM-net secretariat conducts annual telephone interviews to gather clinical information, including HAM/TSP symptoms, quality of life (QOL), living conditions, and treatment status. These surveys have provided valuable insights into the clinical course and management of patients with HAM/TSP [10–12].

The Osame’s Motor Disability Score (OMDS) is widely used in Japan to assess the severity of motor disability and evaluate treatment efficacy in patients with HAM/TSP. The OMDS ranges from 0 to 13, with higher scores indicating greater walking disability. For instance, OMDS scores of 5 and 6 correspond to patients who need unilateral and bilateral support to walk, respectively. The mean disease duration for patients with OMDS scores of 5 and 6 was 15 and 19 years, respectively [12]. The OMDS often serves as the primary endpoint in HAM/TSP clinical trials [13, 14]; however, it frequently requires long-term observation over several years to detect meaningful changes. According to a study using the Japanese HAM-net registry [11], the mean change in OMDS over 1 year was 0.24 in the steroid-treated group (*n* = 131) and 0.26 in the previously steroid-treated group (*n* = 82), with approximately 20% of patients showing an OMDS change of ≥ 1 over the same period in both groups. In contrast, gait speed over a short, fixed distance, such as the 10-meter timed walk (10mTW), is used to assess lower extremity function and reflects gait quality and motor function. A UK cohort study reported a significant correlation between changes in the 10mTW and disease progression [9]. Furthermore, a 6-month prospective study involving patients with HAM/TSP from Japan, the US, the UK, and Brazil suggested that the 10mTW is a feasible outcome measure for clinical trials [15]. Unlike the OMDS, the 10mTW can capture variability in disease progression over shorter observation periods.

In this study, we incorporated 10mTW data from patients with HAM/TSP enrolled in the HAM-net, evaluated the natural progression of 10mTW performance, and analyzed correlations between the 10mTW and other assessment measures, including the OMDS. Our goal was to identify appropriate indicators for the evaluation of treatment efficacy in patients with HAM/TSP.

## Methods

### Study design and population

This observational study analyzed patient data from the HAM-net (jRCT1050220081). The secretariat of the HAM-net study conducts annual telephone interviews to gather clinical information, including HAM/TSP symptoms, QOL, living conditions, and treatment status. Of the 651 patients registered in the HAM-net between April 1, 2012, and March 31, 2022, two analysis populations were defined: the full analysis set (FAS) and 10mTW analysis population. The FAS population comprised patients registered in HAM-net. Conversely, HTLV-1 carriers suspected of having HAM/TSP but lacking a confirmed diagnosis, patients who registered with HAM-net but died before the first telephone interviews to collect clinical information, and patients who failed to complete the annual telephone interviews even once were excluded. The 10mTW analysis population was a subset of the FAS and included patients from the FAS population with at least 2 time points of 10mTW data.

### 10mTW assessment

A stopwatch was used to manually measure the walking test, which was performed following a standardized method as described in the measurement manual. Patients with HAM/TSP were instructed to walk 14 meters, which included a 2-meter initiation phase, a 10-meter walking phase, and a 2-meter termination phase. The time (in seconds) required to walk the 10-meter phase, with or without a walking aid, was recorded. In principle, measurements were performed twice at each assessment, and the average value (rounded to 2 decimal places) was used. Data closest to the first HAM-net interview date were used as baseline. If multiple dates had the same deviation from the interview date, the data before the interview were prioritized. To reduce measurement variability, the same assessor performed the measurements for each patient at each visit whenever possible. Walking assessments were conducted under consistent conditions, with patients either barefoot or wearing shoes if needed, and these conditions were kept the same for each assessment. Furthermore, patients were provided with standardized instructions, with no special encouragement or comments that could influence patients’ motivation.

### Calculations and statistical analysis

The demographic characteristics of both the FAS and 10mTW analysis populations were analyzed. The natural history of the 10mTW and its correlation with other indices were analyzed using the 10mTW analysis population. The specific data collected from the HAM-net are presented in Supplementary Table 1. These indices included the OMDS [10, 14], Instituto de Pesquisa Clinica Evandro Chagas-1 (IPEC-1) [10], HAM-Bladder Dysfunction Severity Grade (HAM-BDSG) [10, 14], HAM-Bladder Dysfunction Symptom Score (HAM-BDSS) [16], Overactive Bladder Symptom Score (OABSS) [10], International Prostate Symptom Score (I-PSS) [10], Health Assessment Questionnaire (HAQ) [10], MOS 36-Item Short-Form Health Survey (SF-36) [10], and Short-Form 6 Dimension (SF-6D) [12]. Pain, foot numbness and overall assessment of HAM condition were assessed using the visual analog scale (VAS). In addition, the modified HAQ for HAM was tabulated excluding HAQ questions 5, 6, 7, 16, and 17 focusing on hand movement since these hand tasks showed very little correlation with motor disability score [10].

Descriptive statistics for the 10mTW were calculated at 1 year before baseline, at baseline, and annually up to 9 years post-baseline. Furthermore, the rate of change from baseline, based on the log-transformed ratio of the 10mTW to baseline, was calculated at each post-baseline assessment point. Point estimates and 95% confidence intervals (CIs) were calculated for the 10mTW at each time point and for the percentage change from baseline.

Spearman’s correlation coefficients were calculated for log-transformed 10mTW values and baseline/post-baseline OMDS, IPEC-1, SF-36 (question 2), and HAM-BDSG scores, and for changes from baseline. Pearson’s and Spearman’s correlation coefficients were calculated for log-transformed 10mTW values and baseline/post-baseline HAQ, modified HAQ for HAM, SF-6D, OABSS, I-PSS, HAM-BDSS, and VAS (pain, numbness, and general condition) scores, and for changes from baseline.

A receiver operating characteristic (ROC) curve analysis was performed using the 10mTW analysis population to calculate the optimal cutoff percentage change in 10mTW that detects disease progression in HAM/TSP, defined as a 1-point worsening in OMDS from baseline versus no change.

All statistical analyses were performed using SAS version 9.4 (SAS Institute Inc., Cary, NC, USA). *p*-values ≤ 0.05 were used to denote statistical significance.

### Ethics approval and consent to participate

The study protocol was approved by the Tanabe Pharma Corporation Human Tissue Research Ethics Review Committee (formerly Mitsubishi Tanabe Pharma Corporation Human Tissue Research Ethics Review Committee) (Approval ID No. H-22–018). Implementation approval for the study was obtained from the president of St. Marianna University School of Medicine (Approval ID No. 5770). This study was conducted according to the Ethical Guidelines for Medical and Biological Research Involving Human Subjects (Ministry of Health, Labour and Welfare, Japan, 2021, revised in 2023), World Medical Association Declaration of Helsinki (revised in 2013), and the Act on the Protection of Personal Information (Japan, Act No. 57 of 2003, last revised in 2021). Because patients registered in HAM-net, which was approved by the Bioethics Committee of St. Marianna University School of Medicine (Approval ID Nos. 2044 and 4417), had provided consent for research participation and secondary use of their data, no additional consent was required for this study.

## Results

### Patient characteristics

**Fig. 1:**
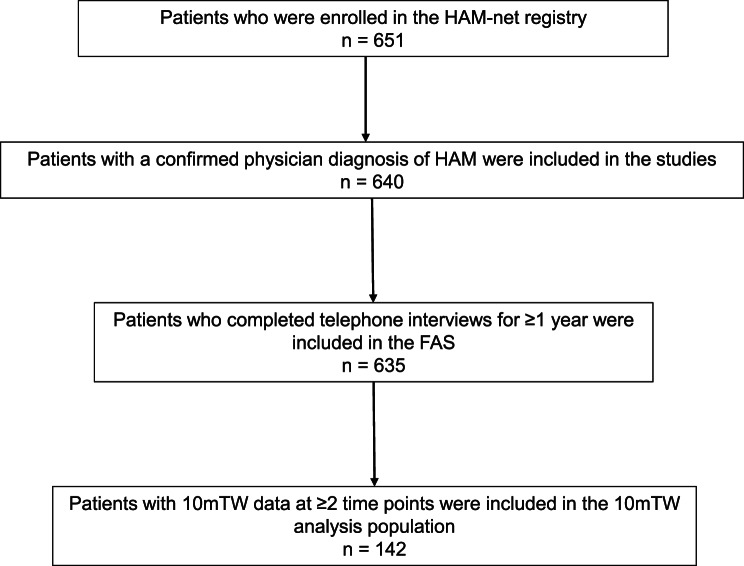
Flowchart showing analysis sets to assess enrolled patients with motor disability. Abbreviations: 10mTW, 10-meter timed walk; FAS, full analysis set; HAM, human T-cell leukemia virus type 1 (HTLV-1)-associated myelopathy

In total, 651 HAM-net registrants were interviewed by telephone for assessment as of the data cutoff date of March 31, 2022. Of these, 11 patients were excluded from the HAM-net study; 9 were HTLV-1 carriers without confirmed HAM/TSP, and 2 died before the HAM-net study began. Among the 640 remaining patients, 635 completed telephone interviews in Year 1, 589 in Year 2, 548 in Year 3, 505 in Year 4, 469 in Year 5, 417 in Year 6, 377 in Year 7, 307 in Year 8, 246 in Year 9, and 196 in Year 10. The 635 patients who completed interviews for at least 1 year were included in the FAS population, and 142 patients with 10mTW data collected at ≥ 2 time points were included in the 10mTW analysis population (Fig. 1).

Table 1 shows the baseline demographic characteristics and treatment histories of the FAS and 10mTW analysis populations. The proportion of female patients was 74.3% and 77.5%, the mean age at onset was 45.8 and 45.7 years, and the mean age at HAM diagnosis was 53.5 and 53.8 years in the FAS and 10mTW analysis populations, respectively. All demographic variables and treatment histories were comparable between the FAS and 10mTW analysis populations, suggesting that the 10mTW analysis population represented patients registered in the HAM-net.

**Table 1:** Baseline demographic and disease characteristics of the participants

	Statistics	FAS (*N* = 635)	10mTW (*N* = 142)
Age at registration, years			
<65	n (%)	335 (52.8)	84 (59.2)
≥65	n (%)	300 (47.2)	58 (40.8)
	Mean ± SD	62.5 ± 11.0	60.2 ± 11.4
	(Min, Max)	(24, 87)	(25, 82)
Sex			
Male	n (%)	163 (25.7)	32 (22.5)
Female	n (%)	472 (74.3)	110 (77.5)
Age at HAM/TSP diagnosis, years			
<65	n (%)	497 (78.3)	107 (75.4)
≥65	n (%)	138 (21.7)	35 (24.6)
	Mean ± SD	53.5 ± 12.8	53.8 ± 12.8
	(Min, Max)	(10, 85)	(16, 82)
Age at HAM/TSP occurrence, years			
Missing	n (%)	3 (0.5)	1 (0.7)
<65	n (%)	564 (88.8)	127 (89.4)
≥65	n (%)	68 (10.7)	14 (9.9)
	n	632	141
	Mean ± SD	45.8 ± 14.9	45.7 ± 14.2
	(Min, Max)	(10, 85)	(15, 81)
Age at occurrence of motor disability, years			
Missing	n (%)	2 (0.3)	0
<65	n (%)	564 (88.8)	130 (91.5)
≥65	n (%)	69 (10.9)	12 (8.5)
	n	633	142
	Mean ± SD	46.9 ± 14.6	47.5 ± 13.3
	(Min, Max)	(10, 85)	(15, 81)
History of medications			
Oral steroid therapy	n (%)	440 (69.3)	107 (75.4)
Methyl-prednisolone pulse therapy	n (%)	263 (41.4)	62 (43.7)
Interferon-α treatment	n (%)	191 (30.1)	30 (21.1)
Medication for urinary dysfunction	n (%)	262 (41.3)	60 (42.3)

Abbreviations: 10mTW, 10-meter timed walk; FAS, full analysis set; HAM/TSP, human T-cell leukemia virus type 1 (HTLV-1)-associated myelopathy/tropical spastic paraparesis; Max, maximum value; Min, minimum value; SD, standard deviation

### Change in the 10mTW

**Fig. 2:**
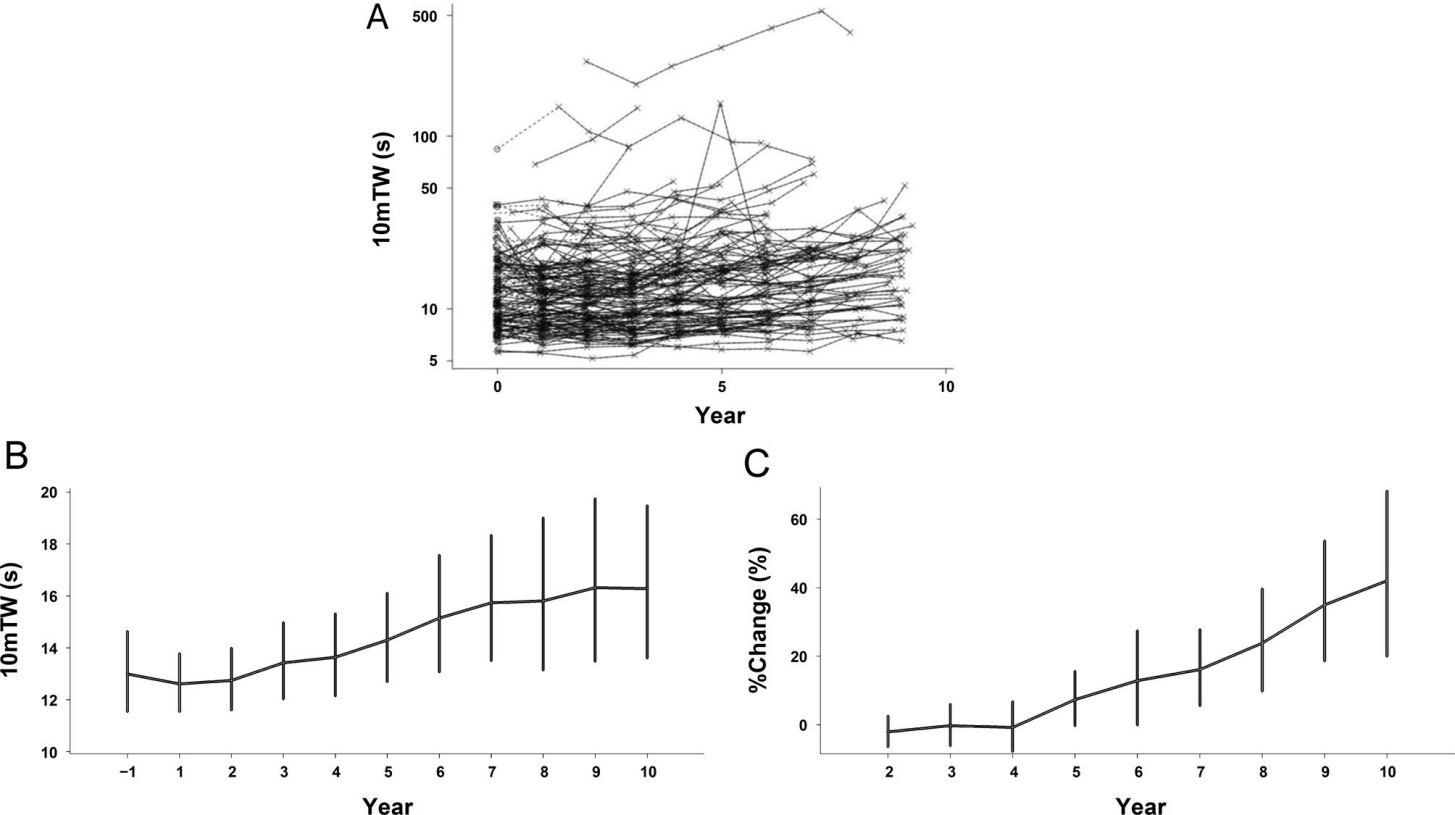
Change in the 10mTW over time. **A**: Individual data; **B**: Geometric mean and 95% CI of the log-transformed 10mTW; **C**: Geometric mean and 95% CI of percent changes in the log-transformed 10mTW from the baseline Abbreviation:10mTW, 10-meter timed walk

The geometric mean of the 10mTW at baseline, Year 5, and Year 10 were 12.6 s (95% CI, 11.5 s–13.8 s), 14.3 s (95% CI, 12.7 s–16.1 s), and 16.3 s (95% CI, 13.6 s–19.5 s), respectively. Compared to baseline, the geometric mean of the 10mTW increased by 7.37% (95% CI, −0.39%–15.73%) at Year 5 and 42.10% (19.92%–68.39%) at Year 10 (Fig. 2).

### Correlation between the 10mTW and outcome measures

**Fig. 3:**
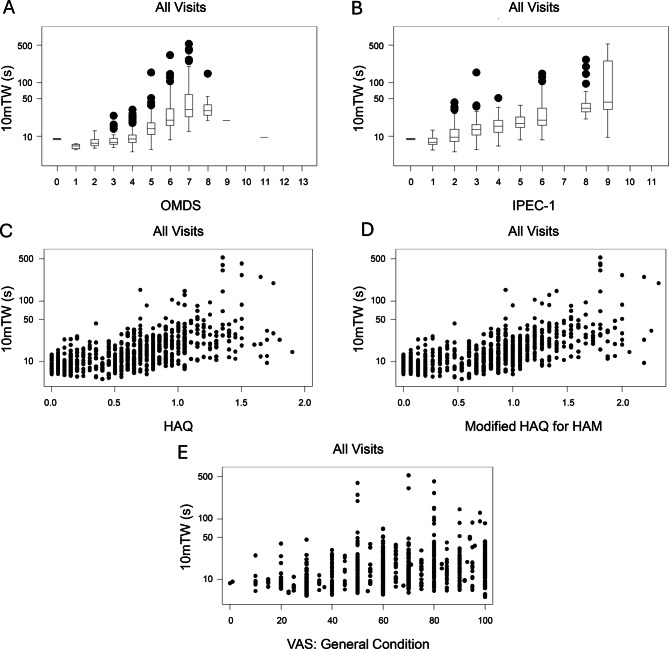
Correlation between the 10mTW and other outcome measures. Box-and-whisker plots of the 10mTW are shown against the Osame’s motor disability score (OMDS) (**A**) and Instituto de Pesquisa Clinica Evandro Chagas-1 (IPEC-1) (**B**). Scattered plots of the 10mTW are shown against Health assessment Questionnaire (HAQ) (**C**), modified HAQ for HTLV-1-associated myelopathy (HAM) (**D**), and visual analog scale (VAS) (general condition) (**E**). Abbreviation:10mTW, 10-meter timed walk

The 10mTW was positively correlated with motor disability scores, such as the OMDS and IPEC-1, and activities of daily living (ADL) scales, such as the HAQ (Fig. 3). Spearman’s correlation coefficients between log-transformed 10mTW values and the OMDS and IPEC-1 were 0.728 (*p* < 0.001) and 0.730 (*p* < 0.001), respectively (Table 2). Pearson’s and Spearman’s correlation coefficients between log-transformed 10mTW values and the HAQ were 0.623 (*p* < 0.001) and 0.666 (*p* < 0.001), respectively. For the modified HAQ for HAM, the corresponding correlation coefficients were 0.652 (*p* < 0.001) and 0.688 (*p* < 0.001) (Table 2). Pearson’s and Spearman’s correlation coefficients between log-transformed 10mTW values and the overall assessment of HAM condition (VAS) were 0.319 (*p* < 0.001) and 0.374 (*p* < 0.001), respectively.

**Table 2:** Correlation between the 10mTW and other outcome measures

Parameter	Pearson’s correlation coefficient	Correlation p-value	Spearman’s correlation coefficient	Correlation p-value
HAQ	0.623	<0.001	0.666	<0.001
Modified HAQ for HAM	0.652	<0.001	0.688	<0.001
SF-6D	−0.251	<0.001	−0.253	<0.001
OABSS	0.058	0.100	0.084	0.017
I-PSS	0.063	0.073	0.081	0.022
HAM-BDSS: Total	0.064	0.071	0.088	0.013
HAM-BDSS: Storage Symptoms	0.039	0.265	0.068	0.054
HAM-BDSS: Voiding Symptoms	0.042	0.219	0.035	0.308
VAS: Pain	0.124	<0.001	0.121	<0.001
VAS: Numbness	0.160	<0.001	0.220	<0.001
VAS: General Condition	0.319	<0.001	0.374	<0.001
OMDS	N/A	N/A	0.728	<0.001
IPEC-1	N/A	N/A	0.730	<0.001
SF-36 (Q2)	N/A	N/A	0.157	0.004
HAM-BDSG	N/A	N/A	0.183	<0.001

Abbreviations: 10mTW, 10-meter timed walk; HAM, human T-cell leukemia virus type 1 (HTLV-1)-associated myelopathy; HAM-BDSG, HAM-Bladder Dysfunction Severity Grade; HAM-BDSS, HAM-Bladder Dysfunction Symptom Score; HAQ, Health Assessment Questionnaire; HTLV-1, human T-cell leukemia virus type 1; IPEC-1, Instituto de Pesquisa Clinica Evandro Chagas-1; I-PSS, International Prostate Symptom Score; OABSS, Overactive Bladder Symptom Score; OMDS, Osame’s Motor Disability Score; SF-36, Medical Outcomes Study 36-Item Short-Form Health Survey; SF-6D, Short-Form 6 dimension; VAS, visual analog scale

### Cutoff percentage change in the 10mTW from baseline

**Fig. 4:**
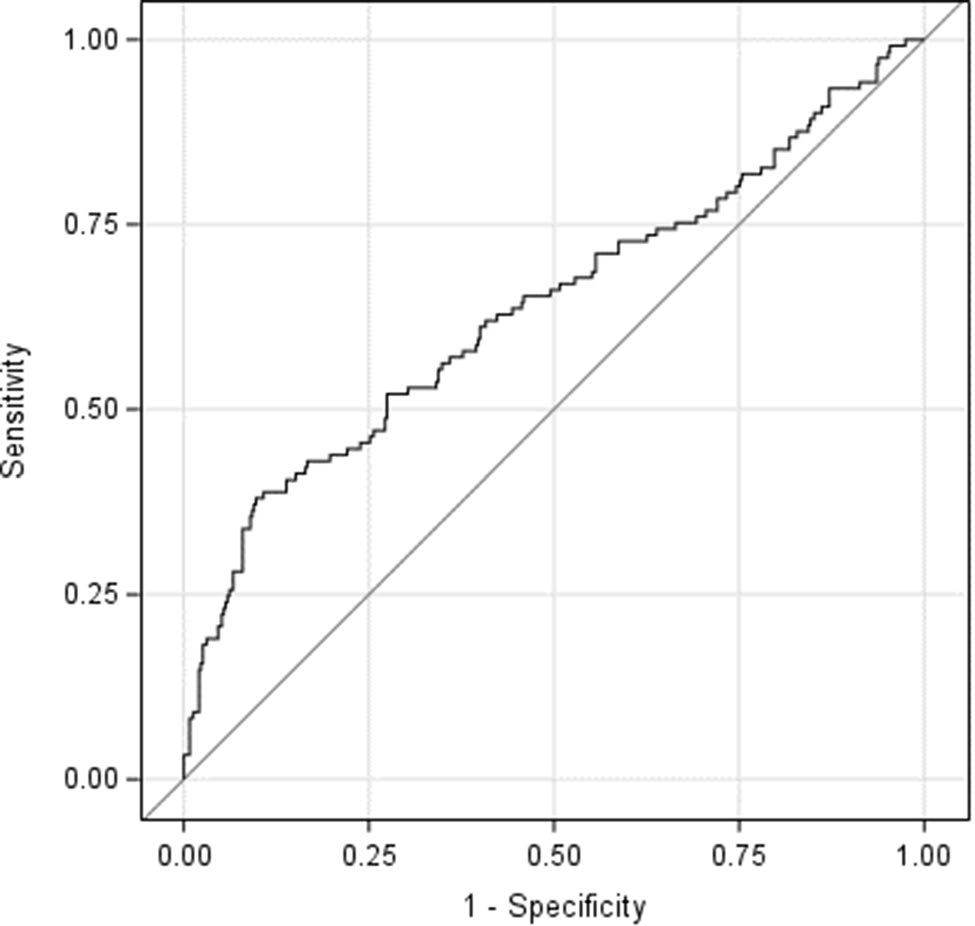
Receiver operating characteristic (ROC) curve for the 10mTW. ROC curve analysis was performed to estimate the optimal cutoff value for the percentage change in 10mTW from baseline that best discriminated between patients whose Osame’s motor disability score (OMDS) worsened by 1 point and those whose OMDS remained stable. The area under the curve (AUC) was 0.6387. Abbreviation: AUC, area under the curve

ROC curve analysis was performed to determine the cutoff percentage change in 10mTW from baseline corresponding to a clinically meaningful 1-point progression in OMDS [11]. The 10mTW demonstrated discriminative ability, with an area under the curve (AUC) of 0.6387 and a cutoff percentage change of >38.42% (95% CI, 21.81%–66.92%; sensitivity, 38.0%; specificity, 90.3%) (Table 3 and Fig. 4).

**Table 3:** Receiver operating characteristic (ROC) analysis for the 10mTW

	AUC	Cutoff point (%) [95% CI]	*p***-value**	Sensitivity (%)	Specificity (%)
10mTW	0.6387	38.42 [21.81, 66.92]	<0.001	38.0	90.3

Abbreviation: AUC, area under the curve; 10mTW, 10-meter timed walk

## Discussion

In this study, we evaluated the long-term progression of 10mTW performance and examined its correlation with clinical assessment indices, such as the OMDS in patients with HAM/TSP. In total, 635 patients who completed telephone interviews for at least 1 year were included in the FAS population, and 142 patients with 10mTW data collected at ≥ 2 time points were included in the 10mTW analysis population. No significant differences in demographic characteristics or treatment history at baseline were observed between the FAS and 10mTW analysis populations, indicating that the 10mTW analysis population represented patients registered in the HAM-net.

Although previous studies have reported a 2–8% decrease in gait speed, corresponding to a 2–9% increase in walking time, from the 60s to 70s among healthy adults [17, 18], the present analysis revealed a significant increase in 10mTW over a 10-year follow-up. Specifically, the geometric mean time increased by 42.10% from baseline to Year 10, indicating substantial disease progression. These findings highlight the progressive nature of HAM/TSP and the clinical relevance of the 10mTW as an outcome measure.

The observed correlation between the 10mTW and established motor disability scores, including the OMDS and IPEC-1, highlights the validity of using the 10mTW as a surrogate marker of motor function. According to the HAM Clinical Practice Guidelines 2019, the OMDS is widely used to assess motor disability, which is a primary symptom of HAM/TSP, and to evaluate treatment efficacy because it reflects disease progression and response to therapy [13]. The OMDS has been shown to correlate with health-related QOL measures, such as the SF-36 and EQ-5D [12, 19]. The present findings confirmed that the 10mTW increases with disease progression, similar to the OMDS. The motor dysfunction items were similar between the OMDS and IPEC-1, and both strongly correlated with the 10mTW. Both OMDS and IPEC-1 include walking ability as a component, which overlaps with the concept of the 10mTW. However, the observed correlation between the 10mTW and OMDS, which reflects broader aspects of patient functioning and disease severity, such as its effect on QOL, as well as IPEC-1, provides evidence supporting the clinical relevance of the 10mTW as a functional measure in HAM/TSP. Furthermore, because the 10mTW is a continuous variable that can detect changes over shorter periods than the OMDS, it is a valuable measure for assessing treatment efficacy in HAM clinical trials.

To the best of our knowledge, this study is the first to estimate a cutoff value for the 10mTW in HAM/TSP. Our results show that the cutoff percentage change in the 10mTW from baseline was 38.42%, as determined by ROC curve analysis. However, this cutoff may represent a greater change than the minimal clinically important difference (MCID) as perceived by patients because a 1-point change in OMDS may correspond to approximately 4–5 years of disease progression in patients with HAM/TSP [11], which is regarded as a change greater than “minimal.” Considering that a 20% decline in walking time is considered a clinically significant threshold in multiple sclerosis, a disease with clinical and pathophysiological similarities to HAM/TSP [20, 21], further studies including ROC curve analysis based on patients’ global impression of severity or changes over shorter duration, such as 1–2 years, are required to determine the MCID for this measure and optimize its use in clinical assessments and trial endpoints.

Moreover, significant correlations with ADL measures such as the HAQ and the modified HAQ for HAM suggest that 10mTW performance reflects not only motor disability but also daily functional capacity. These relationships strengthen the argument for incorporating the 10mTW into clinical trials as a practical, objective, and sensitive endpoint. The HAQ is a commonly used ADL index for chronic rheumatic diseases such as arthritis [22]. A previous study using the HAM-net showed that the HAQ had a strong positive correlation with the OMDS and was a valuable tool for measuring disease severity in HAM/TSP [10]. Furthermore, the modified HAQ for HAM, which excludes items related to upper limb function (questions 5, 6, 7, 16, and 17), was found to be useful for assessing patients with HAM/TSP [10]. In this study, we observed a strong positive correlation between the 10mTW and HAQ, with an even stronger correlation with the modified HAQ, indicating that the 10mTW is closely aligned with ADL indicators. The VAS score of HAM condition, a patient-reported outcome (PRO) that reflects patient perception of HAM-related symptoms at the time of the interview, also showed a positive correlation with the 10mTW, further supporting its relevance as an outcome measure.

In contrast, correlations between the 10mTW and urinary dysfunction indices (i.e., the HAM-BDSG, HAM-BDSS, OABSS, and I-PSS) and sensory dysfunction indices (i.e., pain and numbness on the VAS) were weaker. These findings suggest that gait disturbance progresses independently of urinary or sensory dysfunction.

This study has several limitations. First, the number of participants interviewed at Year 8 and beyond was less than half of those at baseline. This decrease was attributed to variations in follow-up duration among patients because data collection using the HAM-net is ongoing. Therefore, caution is required when interpreting data particularly from later periods. Second, although no significant differences in demographic characteristics and treatment history were observed between the FAS and 10mTW analysis populations, the 10mTW analysis population only included patients who could walk 10 meters and had corresponding data, which may limit the generalizability of these findings. Third, the 10mTW may have been influenced by the addition or modification of walking aids, which was not considered in this study. Fourth, intraindividual variability, such as fatigue and daily fluctuations, was not considered in this study. Furthermore, the 10mTW has inherent limitations, such as relatively low ecological validity, the inability to capture endurance and fatigability, and limited sensitivity to balance disturbances or real-world walking conditions. These factors may limit its value as a stand-alone outcome measure for HAM/TSP, indicating that complementary outcome measures may be necessary for a comprehensive assessment of disease progression. Despite these limitations, the large sample size and long-term follow-up provide valuable insights into HAM/TSP progression. Future research should explore factors influencing the variability in gait decline, including disease duration, treatment history, and comorbid conditions. Furthermore, the responsiveness of the 10mTW to therapeutic interventions could establish its use in assessing treatment efficacy.

## Conclusions

In conclusion, this study demonstrated that the 10mTW gradually increases over time and is strongly correlated with motor disability scores, ADL scales, and PRO measures used to assess treatment efficacy, suggesting that the 10mTW is a reliable and meaningful clinical endpoint reflecting motor function and ADL in patients with HAM/TSP. These findings support the use of the 10mTW as a primary outcome measure in future HAM/TSP clinical trials, and future studies should focus on determining the minimal clinically important difference for this measure to optimize its use in clinical assessments and trial endpoints.

## Electronic supplementary material

Below is the link to the electronic supplementary material.

Supplementary material 1

## Data Availability

The data may be shared upon reasonable request to the corresponding author.

## Abbreviations

10mTW: 10-meter timed walk
ADL: Activities of daily living
CI: Confidence interval
HAM: HTLV-1-associated myelopathy
HAM-BDSG: HAM-Bladder dysfunction severity grade
HAM-BDSS: HAM-Bladder dysfunction symptom score
HAM-net: National registration system for patients with HAM
HAQ: Health assessment questionnaire
HTLV-1: Human T-cell leukemia virus type 1
IPEC-1: Instituto de Pesquisa Clinica Evandro Chagas-1
I-PSS: International prostate symptom score
OABSS: Overactive bladder symptom score
OMDS: Osame’s motor disability score
PRO: Patient-reported outcome
SF-36: Medical Outcomes Study 36-item short-form health survey
SF-6D: Short-form 6 dimension
TSP: Tropical spastic paraparesis
VAS: Visual analog scale
