# Supplementary material for: Use of the 10-meter timed walk for monitoring long-term gait progression in human T-cell leukemia virus type 1-associated myelopathy/tropical spastic paraparesis: a 10-year analysis from the Japanese HAM-net registry
Source: Orphanet J Rare Dis. 2026 May 25;21:249. doi: 10.1186/s13023-026-04399-y (PMC13390377; doi:10.1186/s13023-026-04399-y)
Supplement: Supplementary file 1 — Supplementary material 1 [file 13023_2026_4399_MOESM1_ESM.docx]

**Supplementary Table 1. Parameters analyzed**

| Parameter | Description |
| --- | --- |
| Patient background | Sex |
|  | Year of birth |
| HAM/TSP symptoms | Time of diagnosis |
|  | Initial symptoms and time of onset |
|  | Information on walking aids |
|  | Information on urinary and excretory disorders |
| Indicators for evaluating HAM/TSP symptoms | |
| Osame’s Motor Disability Score (OMDS) | Score range: 0–13, with higher scores indicating greater walking disability |
| Instituto de Pesquisa Clinica Evandro Chagas-1 (IPEC-1) | Score range: 0–29 covering motor, spasticity, sensory, and sphincter functions, with higher scores indicating greater disability |
| HAM-Bladder Dysfunction Severity Grade (HAM-BDSG) | Score range: 0–13, with higher scores indicating greater walking disability |
| HAM-Bladder Dysfunction Symptom Score (HAM-BDSS) | Total score range: 0–40, based on selected questions from the International Prostate Symptom Score [I-PSS] and Overactive Bladder Symptom Score with higher scores indicating more severe bladder symptoms |
| Overactive Bladder Symptom Score (OABSS) | Total score range: 0–15, based on 4 questions, with higher scores indicating greater urinary urgency |
| International Prostate Symptom Score (I-PSS) | Total score range: 0–35, based on 7 questions, with higher scores indicating greater difficulty urinating |
| Health Assessment Questionnaire (HAQ) | Average of scores of 0–3 across 20 items, with higher scores indicating difficulty in performing daily tasks |
| Modified HAQ for HAM | Average of scores of 0–3 across 20 items, excluding questions 5, 6, 7, 16, and 17 of HAQ |
| Question 2 of MOS 36-Item Short-Form Health Survey (SF-36) | Average score set at 50 for Japanese population, with higher scores indicating better health-related quality of life |
| Short-Form 6 Dimension (SF-6D) | A health index |
| Information on pain | Including Visual Analog Scale (VAS) |
| Information on foot numbness | Including VAS |
| Overall assessment of HAM condition | Including VAS |
| Treatment history | Oral steroids |
|  | Pulse steroid therapy |
|  | Interferon alpha |
|  | Medication status related to dysuria |
|  | Details of exercise therapy and rehabilitation |
| Others | Information on complications and medical history |

Abbreviation: HAM/TSP, Human T-cell leukemia virus type 1 (HTLV-1)-associated myelopathy/tropical spastic paraparesis
